# Supplementary material for: Ambulatory specialist costs and morbidity of coordinated and uncoordinated patients before and after abolition of copayment: A cohort analysis
Source: PLoS One. 2021 Jun 28;16(6):e0253919. doi: 10.1371/journal.pone.0253919 (PMC8238183; doi:10.1371/journal.pone.0253919)
Supplement: S1 Fig — (PDF) [file pone.0253919.s002.pdf]

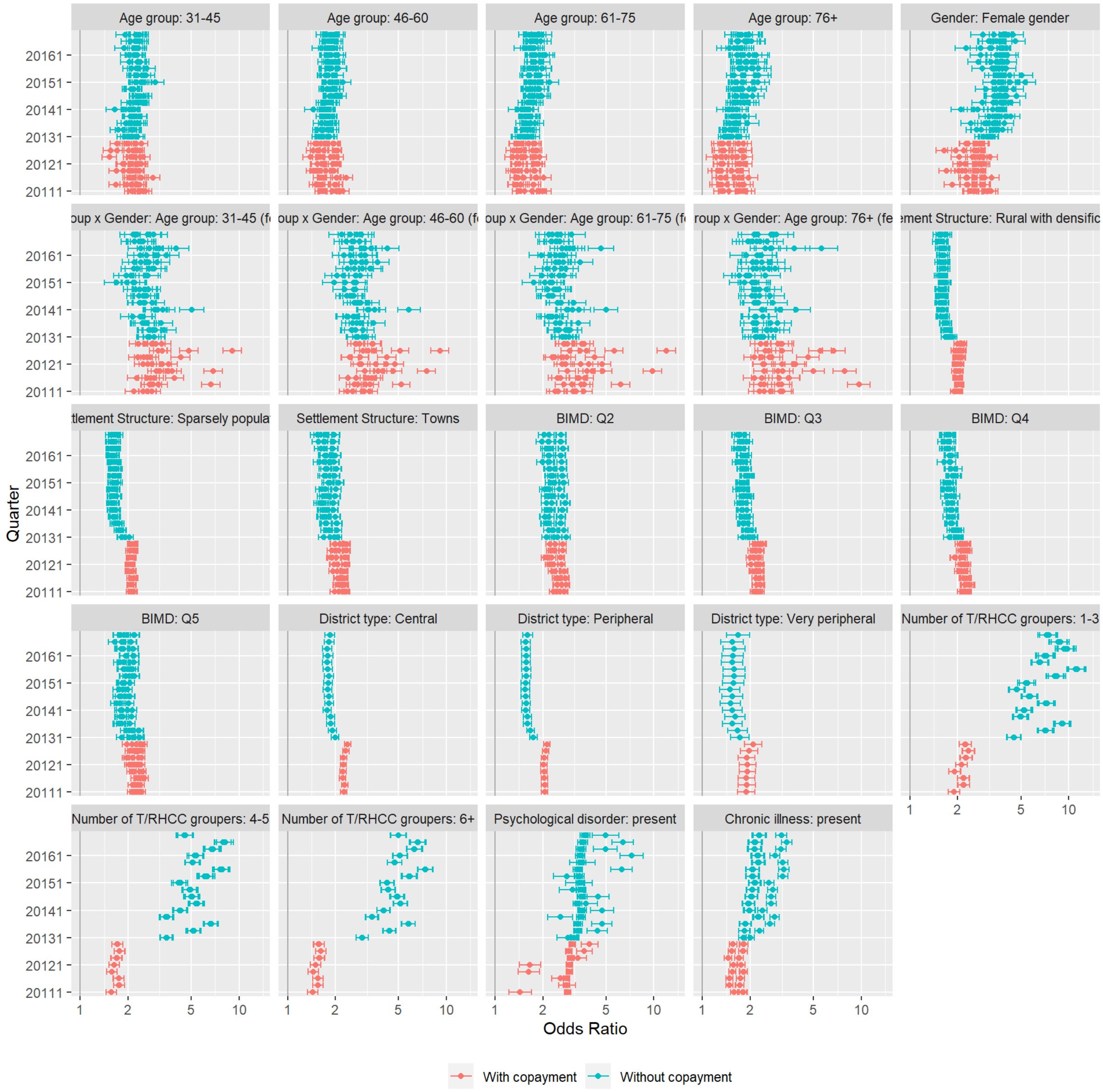

S1 Fig. Longitudinal regression modelling (generalised estimating equations, GEE), comparing uncoordinated patients (UP) vs. coordinated patients (CP) (Odds Ratio, 95%-CI).

Reference: age: 18-30; age x sex: 18-30 (male); BIMD: 1st BIMD quintile (lowest deprivation); presence of chronic illness: no; presence of psychological disorder: no; sex: male; settlement structure: urban; district type: very central; number of diagnosis groups: [0, 1].

In S1 Fig, a higher OR represents a higher degree of uncontrolled specialist contacts (UP), whereas a smaller OR represents a higher degree of GP-coordinated specialist contacts (CP). The different thickness of the lines results from the parallel presentation of all models, which were built up successively (see also S1 Table). A large number of model estimates are quiet similar, so the estimates are overlapping. As a result, the lines appear thicker, due to very narrow, overlapping confidence intervals.
