# Supplementary material for: Ambulatory specialist costs and morbidity of coordinated and uncoordinated patients before and after abolition of copayment: A cohort analysis
Source: PLoS One. 2021 Jun 28;16(6):e0253919. doi: 10.1371/journal.pone.0253919 (PMC8238183; doi:10.1371/journal.pone.0253919)
Supplement: S2 Fig — Distribution of probabilities to belong to the coordination subgroups coordinated patients (CP) (a), uncoordinated patients (UP) (b), or GP care only (GP) (c), quarterly. (PDF) [file pone.0253919.s003.pdf]

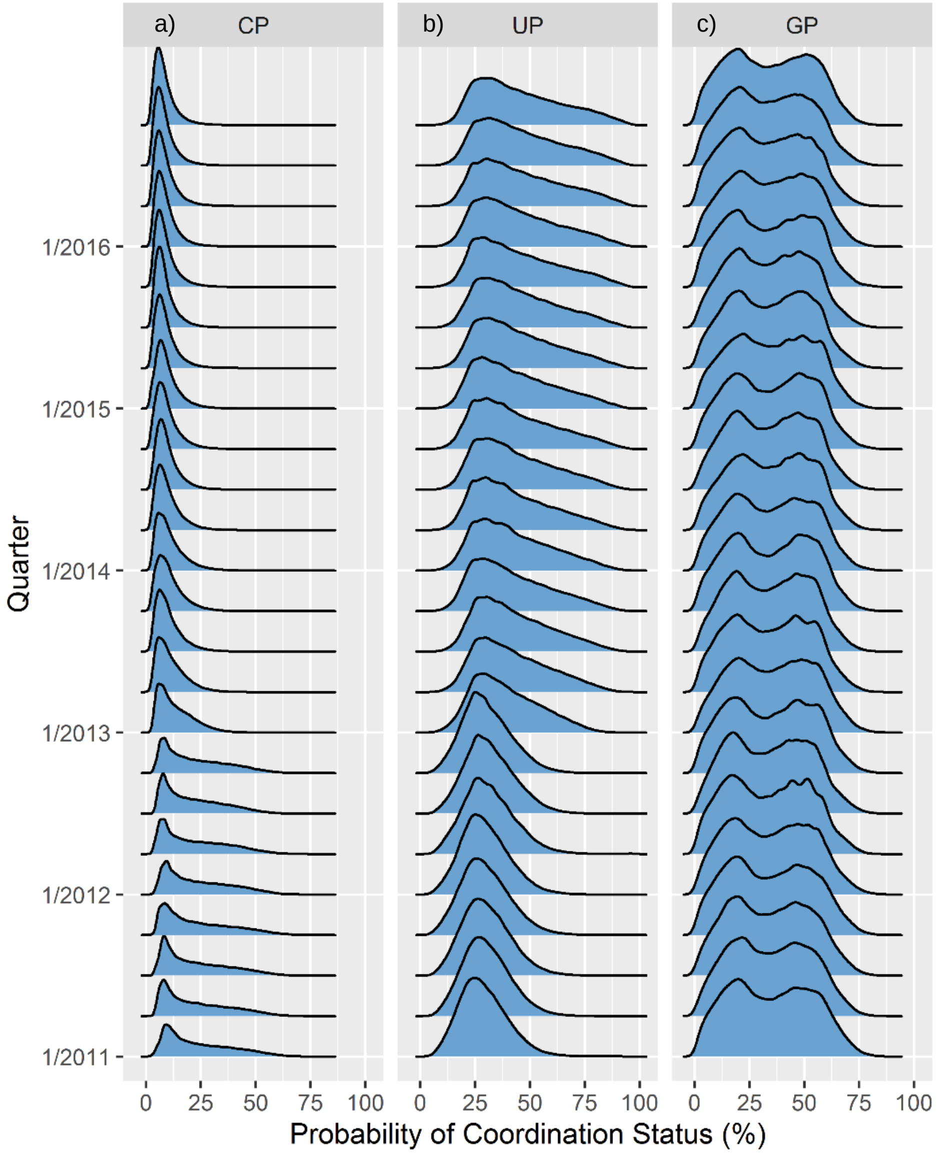

S2 Fig. Distribution of probabilities to belong to the coordination subgroups coordinated patients (CP) (a), uncoordinated patients (UP) (b), or GP care only (GP) (c), quarterly.

Based on multinomial regression models (see also S1 Table), it was estimated which probability each patient with given characteristics (age, sex, morbidity, and residency) has of belonging to one of the three coordination subgroups (CP, UP, GP) (S2 Fig). Regarding the probability distribution over time, again CP and UP showed the greatest changes comparing the situations with and without co-payment. In terms of CP (a), a wide range of probabilities was observed before abolition, but immediately after 2012 this spectrum condensed and the probabilities now cumulated at a much lower level.

In contrast, the UP group showed an opposite trend (b). During the co-payment, the probability of belonging to UP was 10 to 55%. Conversely, the probability spectrum after 2012 was much broader and a bit higher overall (~20-90%).

The subgroup GP care only (c) remained relatively stable over time. The presentation of GP patients indicated that two probability areas occur more frequently (double peak distribution) and a great proportion of patients had a mean probability to have only contact with general practitioner within a quarter, irrespective of the copayment abolition.
