# Supplementary material for: Ambulatory specialist costs and morbidity of coordinated and uncoordinated patients before and after abolition of copayment: A cohort analysis
Source: PLoS One. 2021 Jun 28;16(6):e0253919. doi: 10.1371/journal.pone.0253919 (PMC8238183; doi:10.1371/journal.pone.0253919)
Supplement: S3 Fig — Outcome: Ambulatory specialist costs. (PDF) [file pone.0253919.s004.pdf]

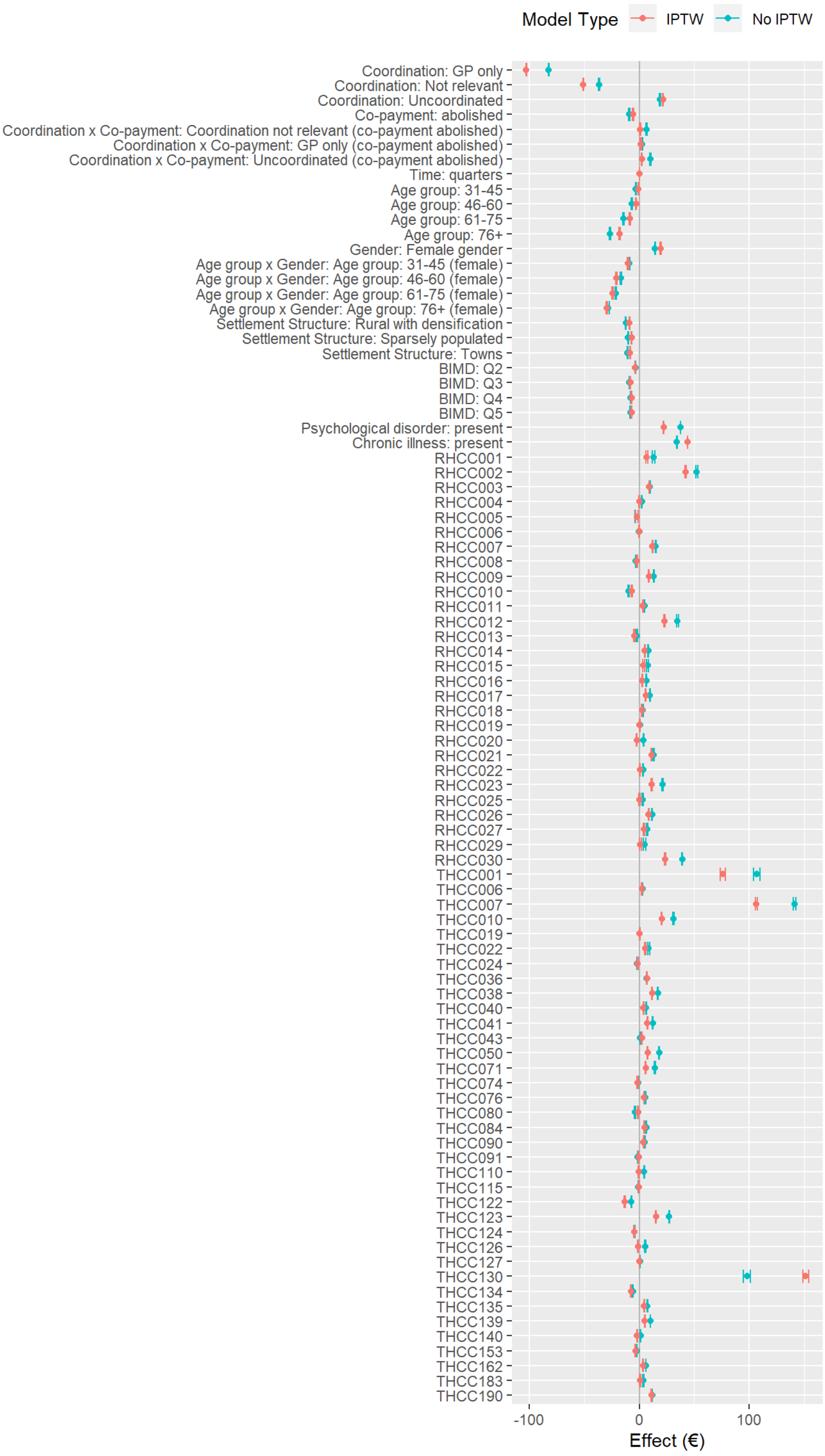

S3 Fig. Longitudinal regression modelling (generalised estimating equations, GEE) with (red) and without (blue) weights for coordination (Inverse Probability of Treatment Weighting, IPTW). Outcome: Ambulatory specialist costs (in €; 95%-CI).

Reference: coordination: coordinated; co-payment: present; coordination x co-payment: coordinated (co-payment present); t (time): quarter 1/2011; age group: 18-30; sex: male; age group x gender: 18-30 (male); settlement structure: urban; BIMD: 1st BIMD quintile (lowest deprivation); psychological disorder: not present; chronic illness: not present; THCC/RHCC: not present.

S3 Fig represents a graphical illustration of Table 3 in the main manuscript. In contrast to Table 3, the results are shown with weights (red) and without weights (blue), whereas Table 3 in the main manuscript contains only weighted cost estimations.
In addition, S3 Fig also presents the single medical condition categories (THCC/RHCC). In order to improve readability, these are not listed in the main manuscript.
The successive model structure which was used to estimate the ambulatory specialist costs, is presented in S2 Table. The presented model represents the full model (Model-No. 15).
