# Supplementary material for: Ambulatory specialist costs and morbidity of coordinated and uncoordinated patients before and after abolition of copayment: A cohort analysis
Source: PLoS One. 2021 Jun 28;16(6):e0253919. doi: 10.1371/journal.pone.0253919 (PMC8238183; doi:10.1371/journal.pone.0253919)
Supplement: S1 Table — Outcome: Coordination status (CP, UP, GP, NR). (PDF) [file pone.0253919.s005.pdf]

S1 Table. Successive model structure used to determine the probability of coordination. Outcome: Coordination status (CP, UP, GP, NR).

**Model-No.**	**Formula**
Basic model, interaction between age and sex	
1	age (cat.) * sex
Regional structure	
2	age (cat.) + sex + settlement structure
3	age (cat.) + sex + BIMD (quintile)
4	age (cat.) + sex + district type
Morbidity (aggregated)	
5	age (cat.) + sex + settlement structure + number of medical condition categories + presence of psychological disorder + presence of chronic illness
6	age (cat.) + sex + BIMD (quintile) + number of medical condition categories + presence of psychological disorder + presence of chronic illness
7	age (cat.) * sex + settlement structure + BIMD (quintile) + number of medical condition categories + presence of psychological disorder + presence of chronic illness
Detailed morbidity (single THCC/RHCC diagnoses)	
8	age (cat.) * sex + THCC/RHCC (70[1] categories)
9	age (cat.) * sex + settlement structure + BIMD (quintile) + THCC/RHCC (70 categories)

Reference: age: 18-30; sex: male; age x sex: 18-30 (male); settlement structure: urban; BIMD: 1st BIMD quintile (lowest deprivation); presence of chronic illness: no; presence of psychological disorder: no; district type: very central; number of diagnosis groups: [0, 1]; THCC/RHCC: diagnosis not present.

Note: Coordination categories: CP: coordinated patient (specialist contact with referral); UP: uncoordinated patient (specialist contact without referral); GP: General practitioner care only (no specialist contact); NR: not relevant for coordinated care.

[1] 70 categories: Due to lack of relevance, two of the 72 diagnosis groups were excluded from modelling:
- RHCC028: Diseases and conditions of a newborn
- RHCC031: Other symptoms, signs of disease, disorders and contact causes without the presence of diagnoses outside the ACC031
