# Supplementary material for: Ambulatory specialist costs and morbidity of coordinated and uncoordinated patients before and after abolition of copayment: A cohort analysis
Source: PLoS One. 2021 Jun 28;16(6):e0253919. doi: 10.1371/journal.pone.0253919 (PMC8238183; doi:10.1371/journal.pone.0253919)
Supplement: S2 Table — (PDF) [file pone.0253919.s006.pdf]

S2 Table. Successive model structure used to estimate ambulatory specialist costs (in €).

Model-No.	Formula
Basic model, without adjustments for patient characteristics/regional structure	
1	t + coordination + co-payment abolished
2	t + coordination * co-payment abolished
Age and sex	
3	t + coordination * co-payment abolished + age (cat.) + sex
4	t + coordination * co-payment abolished + age (cat.) * sex
Regional structure	
5	t + coordination + co-payment abolished + age (cat.) * sex + settlement structure
6	t + coordination + co-payment abolished + age (cat.) * sex + BIMD (quintile)
7	t + coordination + co-payment abolished + age (cat.) * sex + BIMD (quintile) * settlement structure
8	t + coordination + co-payment abolished + age (cat.) * sex + BIMD (quintile) + district type
Morbidity (aggregated)	
9	t + coordination + co-payment abolished + age (cat.) * sex + number of medical condition categories
10	t + coordination + co-payment abolished + age (cat.) * sex + presence of psychological disorder + presence of chronic illness
11	t + coordination + co-payment abolished + age (cat.) * sex + number of medical condition categories + presence of psychological disorder + presence of chronic illness
12	t + coordination * co-payment abolished + age (cat.) * sex + settlement structure + BIMD (quintile) + number of medical condition categories + presence of psychological disorder + presence of chronic illness
Detailed morbidity (single THCC/RHCC diagnoses)	
13	t + coordination * co-payment abolished + age (cat.) * sex + THCC/RHCC (70[1] categories)
14	t + coordination * co-payment abolished + age (cat.) * sex + presence of psychological disorder + presence of chronic illness + THCC/RHCC (70 categories)
15	t + coordination * co-payment abolished + age (cat.) * sex + presence of psychological disorder + presence of chronic illness + settlement structure + BIMD (quintile) + THCC/RHCC (70 categories)

Reference: t (time): quarter 1/2011; coordination: coordinated (CP); co-payment: present; age: 18-30; sex: male; age x sex: 18-30 (male); settlement structure: urban; BIMD: 1st BIMD quintile (lowest deprivation); presence of chronic illness: no; presence of psychological disorder: no; district type: very central; number of diagnosis groups: [0, 1]; THCC/RHCC: diagnosis not present.

[1] 70 categories: Due to lack of relevance, two of the 72 diagnosis groups were excluded from modelling:
- RHCC028: Diseases and conditions of a newborn
- RHCC031: Other symptoms, signs of disease, disorders and contact causes without the presence of diagnoses outside the ACC031
