# Supplementary material for: Ambulatory specialist costs and morbidity of coordinated and uncoordinated patients before and after abolition of copayment: A cohort analysis
Source: PLoS One. 2021 Jun 28;16(6):e0253919. doi: 10.1371/journal.pone.0253919 (PMC8238183; doi:10.1371/journal.pone.0253919)
Supplement: S3 Table — (PDF) [file pone.0253919.s007.pdf]

S3 Table. Characteristics of the cohort at the time of the copayment (2012) and after its abolition (2014), divided into coordination status (CP, UP, GP, NR) (only 1st quarters presented).

	Quarter	Total	CP	UP	GP	NR
**Number of patients (%)**	1/2012	502 542	114 867 (22.9)	152 274 (30.3)	174 148 (34.7)	61 253 (12.2)
	1/2014	489 904	52 185 (10.7)	209 419 (42.7)	170 284 (34.8)	58 016 (11.8)
**Age (mean)**	1/2012	49.3	56.1	49.0	48.7	39.2
	1/2014	49.0	55.8	50.5	48.6	39.2
**Gender: female (%)**	1/2012	56.2	58.0	55.8	47.2	79.4
	1/2014	56.2	52.1	58.2	47.5	78.5
**Proportion with residence 'city' (%)**	1/2012	44.7	43.3	49.2	41.6	44.7
	1/2014	44.5	36.6	49.1	41.2	44.9
**Number of medical condition categories/ patient (mean)**	1/2012	5.6	8.2	5.8	4.3	4.0
	1/2014	6.0	7.7	7.2	4.7	4.4
**Number of cases/patient (mean)**	1/2012	2.2	3.5	2.7	1.0	1.9
	1/2014	2.3	3.1	3.2	1.1	2.0
**Proportion with chronic diseases (%)**	1/2012	57.2	80.3	54.0	52.0	37.0
	1/2014	61.5	75.2	67.8	56.5	41.3
**Proportion with mental diseases (%)**	1/2012	28.1	40.5	30.2	20.4	21.2
	1/2014	30.5	34.9	37.9	22.5	23.3
**Specialist financial claims in € (mean)**	1/2012	87.4	138.1	153.3	2.3	70.4
	1/2014	94.8	130.0	166.4	2.8	75.1
**General practitioner financial claims in € (mean)**	1/2012	48.8	73.3	38.3	47.6	32.7
	1/2014	50.0	63.7	51.7	48.5	36.0

Note: Coordination categories: CP: coordinated patient (specialist contact with referral); UP: uncoordinated patient (specialist contact without referral); GP: General practitioner care only (no specialist contact); NR: not relevant for coordinated care.

S3 Table corresponds to Table 1 of the main manuscript, extended by a quarter after the abolition. As the amount of cohort drop-outs increased in the end of the observation period, 2014 (not 2016) was chosen in order to reach a better comparability.
