# Supplementary material for: Ambulatory specialist costs and morbidity of coordinated and uncoordinated patients before and after abolition of copayment: A cohort analysis
Source: PLoS One. 2021 Jun 28;16(6):e0253919. doi: 10.1371/journal.pone.0253919 (PMC8238183; doi:10.1371/journal.pone.0253919)
Supplement: Attachment — Submitted filename: Response to Reviewers.pdf [file pone.0253919.s008.pdf]

Response to Reviewers

Manuscript ID: PONE-D-21-02946

"Ambulatory specialist costs and morbidity of coordinated and uncoordinated patients before and after abolition of copayment: A cohort analysis"

Dear editor, dear reviewers,

Thank you for reviewing our manuscript. Your comments were very constructive and we believe that this critical reflection has improved our manuscript. We have tried to answer your comments appropriately. In the following, we address your comments point by point and highlight changes we have made to the text (line specifications refer to track change version).

Journal Requirements:

When submitting your revision, we need you to address these additional requirements.

1. Please ensure that your manuscript meets PLOS ONE's style requirements, including those for file naming. The PLOS ONE style templates can be found at

https://journals.plos.org/plosone/s/file?id=wjVg/PLOSOne_formatting_sample_main_body.pdf and

https://journals.plos.org/plosone/s/file?id=ba62/PLOSOne_formatting_sample_title_authors_affiliations.pdf

R: Thank you for the style templates. We have adjusted following parts:

- Commas in the author descriptions have been moved to the end, e.g. "Michaela Olm,[1¶*]" is now "Michaela Olm[1¶*],"
- States were added to the author descriptions
- The designations of equal contributorship have been deleted from the title page. The detailed contribution of each author is now listed in the section "Author Contributions", as we believe that this reflects the level of participation more adequately.
- We added ".tif" to each file naming, e.g. "Fig1" is now "Fig1.tif"
- The format of supporting information citations was changed. "Table S1", "Table S2",… or "Fig S1"… are now cited in single files as "S1 Table" , "S2 Table"… or "S1 Fig" and include the designation ".pdf".

2. Please review your reference list to ensure that it is complete and correct. If you have cited papers that have been retracted, please include the rationale for doing so in the manuscript text, or remove these references and replace them with relevant current references. Any changes to the reference list should be mentioned in the rebuttal letter that accompanies your revised manuscript. If you need to cite a retracted article, indicate the article's retracted status in the References list and also include a citation and full reference for the retraction notice.

R: We have carefully checked our reference list. All articles are still available and none have been retracted. We noticed that the article by Brenner et al. (2005) can only be found via the German title, although the journal "Zeitschrift für Allgemeinmedizin" provides an English title

(but is not indexed in Medline). To enable fast identification of the cited article, the reference has been changed. We now use the German title: “Brenner G, Koch H, Franke A. Steuert die Praxisgebühr in die richtige Richtung? - Analyse des Versorgungsgeschehens nach Einführung der „Praxisgebühr”. Z Allg Med. 2005;81(09):377-81.”

Furthermore, the homepage of the BBSR has received a new launch in June 2020 and the cited link is unfortunately no more available. We have identified the new link to the cited content and updated the reference, from “BBSR. Laufende Raumbeobachtung - Raumabgrenzungen. Siedlungsstrukturelle Kreistypen 2017 [Cited 2020 May 28]. Available from: https://www.bbsr.bund.de/BBSR/DE/Raumbeobachtung/Raumabgrenzungen/deutschland/kreise/Kreistypen4/kreistypen_node.html.” to “BBSR. Raumabgrenzungen: Referenzdateien. [Cited 2021 June 8]. Available from https://www.bbsr.bund.de/BBSR/DE/forschung/raumbeobachtung/downloads/download-referenzen.html”

3. We note that you have indicated that data from this study are available upon request. PLOS only allows data to be available upon request if there are legal or ethical restrictions on sharing data publicly. For information on unacceptable data access restrictions, please see http://journals.plos.org/plosone/s/data-availability#loc-unacceptable-data-access-restrictions.

In your revised cover letter, please address the following prompts:

a) If there are ethical or legal restrictions on sharing a de-identified data set, please explain them in detail (e.g., data contain potentially identifying or sensitive patient information) and who has imposed them (e.g., an ethics committee). Please also provide contact information for a data access committee, ethics committee, or other institutional body to which data requests may be sent.

b) If there are no restrictions, please upload the minimal anonymized data set necessary to replicate your study findings as either Supporting Information files or to a stable, public repository and provide us with the relevant URLs, DOIs, or accession numbers. Please see http://www.bmj.com/content/340/bmj.c181.long for guidelines on how to de-identify and prepare clinical data for publication. For a list of acceptable repositories, please see http://journals.plos.org/plosone/s/data-availability#loc-recommended-repositories.

We will update your Data Availability statement on your behalf to reflect the information you provide.

R: Thank you for your note. We hope to meet your requirements by reformulating our Data Availability statement: “The data that support the findings of this study are held by the Bavarian Association of Statutory Health Insurance Physicians. The data are not publically available due to data protection regulations, but may be made available to researchers upon reasonable request and with the permission of the Bavarian Association of Statutory Health Insurance Physicians (E-mail: versorgungsforschung@kvb.de).”

4. Thank you for stating the following in the Competing Interests section:

"I have read the journal's policy and the authors of this manuscript have the following competing interests: ED, MT and RG are employees of the Bavarian Association of Statutory Health Insurance Physicians.

The authors MO, KL, WM, LS and AS declare that they have no competing interests."

Please confirm that this does not alter your adherence to all PLOS ONE policies on sharing data and materials, by including the following statement: "This does not alter our adherence to PLOS ONE policies on sharing data and materials.” (as detailed online in our guide for authors http://journals.plos.org/plosone/s/competing-interests). If there are restrictions on sharing of data and/or materials, please state these. Please note that we cannot proceed with consideration of your article until this information has been declared.

Please include your updated Competing Interests statement in your cover letter; we will change the online submission form on your behalf.

R: We have updated our Competing Interests section: "I have read the journal's policy and the authors of this manuscript have the following competing interests: ED, MT and RG are employees of the Bavarian Association of Statutory Health Insurance Physicians. This does not alter our adherence to PLOS ONE policies on sharing data and materials. The authors MO, KL, WM, LS and AS declare that they have no competing interests."

Please know it is PLOS ONE policy for corresponding authors to declare, on behalf of all authors, all potential competing interests for the purposes of transparency. PLOS defines a competing interest as anything that interferes with, or could reasonably be perceived as interfering with, the full and objective presentation, peer review, editorial decision-making, or publication of research or non-research articles submitted to one of the journals. Competing interests can be financial or non-financial, professional, or personal. Competing interests can arise in relationship to an organization or another person. Please follow this link to our website for more details on competing interests: http://journals.plos.org/plosone/s/competing-interests

Reviewers' comments:

Reviewer's Responses to Questions

Comments to the Author

R: In order to improve readability, questions without reviewer comments (or with answer “Yes”) have been removed (1., 2., and 6.).

3. Have the authors made all data underlying the findings in their manuscript fully available?

The PLOS Data policy requires authors to make all data underlying the findings described in their manuscript fully available without restriction, with rare exception (please refer to the Data Availability Statement in the manuscript PDF file). The data should be provided as part of the manuscript or its supporting information, or deposited to a public repository. For example, in addition to summary statistics, the data points behind means, medians and variance measures should be available. If there are restrictions on publicly sharing data—e.g. participant privacy or use of data from a third party—those must be specified.

Reviewer #1: Yes

Reviewer #2: No

R: For data protection reasons, access to data is restricted. The data holder, the Bavarian Association of Statutory Health Insurance Physicians (BASHIP), is able to provide data access for research purposes. Reasonable requests for data access or additional analysis will be considered by the BASHIP in consultation with its scientific advisory board and data protection

officer. In connection with previous studies, the authors have for example provided additional data to assist with interpretation.

We have now reformulated the Data Sharing Statement as follows:

“The data that support the findings of this study are held by the Bavarian Association of Statutory Health Insurance Physicians. The data are not publically available due to data protection regulations, but may be made available to researchers upon reasonable request and with the permission of the Bavarian Association of Statutory Health Insurance Physicians (E-mail: versorgungsforschung@kvb.de). ”

4. Is the manuscript presented in an intelligible fashion and written in standard English?

PLOS ONE does not copyedit accepted manuscripts, so the language in submitted articles must be clear, correct, and unambiguous. Any typographical or grammatical errors should be corrected at revision, so please note any specific errors here.

Reviewer #1: No

Reviewer #2: Yes

5. Review Comments to the Author

Please use the space provided to explain your answers to the questions above. You may also include additional comments for the author, including concerns about dual publication, research ethics, or publication ethics. (Please upload your review as an attachment if it exceeds 20,000 characters)

Reviewer #1: The manuscript needs English proofreading.

R: One of the authors (ED) is native English speaker. He carefully checked the revised version. Corrections are marked in the manuscript.

Reviewer #2: This manuscript deals with an interesting topic. It is well-written and well-structured, and can have a contribution to the international literature on the subject.

The Introduction is concise and informative, while the data and methods are very detailed and transparent.

The results are well-presented and discussed, while the manuscript also includes a concise discussion of the limitations of this work.

Overall, I do not see any major issues with this manuscript.

R: Thank you very much for your supportive comments.

There is one clarification I would like to see. The authors state that “We performed a retrospective routine data analysis, using claims data of the Bavarian Association of the Statutory Health Insurance Physicians during the period 2011–2012 (with copayment) and 2013–2016 (without copayment), covering 24 quarters.”.

Why are data before 2011 not used? Meaning, why not split the examined period in two equal periods (in terms of number of years)?

R: There were two reasons for starting the observation in the year 2011. First, GP claims data before this point are incomplete for the years 2009 and 2010 because widespread GP-centred contracts were billed via a third-party. After 2011, fewer patients were enrolled in the

programmes and the ICD-10 diagnoses of the enrolled patients are available in most cases. Second, breaks in the underlying data make it difficult to perform a comparable analysis before the year 2010.

The following sentence was added to section “Study design”: “Due to limitations in the data quality with respect the present analysis, time periods before 2011 were not considered.” (see lines 102-104)

Moreover, in the “Cohort size” it is stated that “Between 2012 and 2016, the number of cohort participants decreased by approximately 10%....”. This is a bit confusing and needs rephrasing, as it could be read like the period starts in 2012 and not in 2011.

R: We apologize for the confusing formulation. We have corrected the sentence “Between 2011 and 2016,…”. (see line 261)

Finally, I believe that the Figures should be modified in order to increase their appearance. Also, is there any sort of, e.g., heat map or other form of visualization that could be included in the analysis?

R: Thank you for your suggestion. We have added a line in Figures 1-3, which highlights the date of the copayment abolition. The following sentence was added to the figure legends: “vertical line marks the point of abolition”.

We hope that this additional information contributes to a concise presentation. While a heat map can be a very helpful for of communication, we consider that conventional line charts best facilitate simple and accurate comprehension of the time series.
